# Biological characteristics and metabolic phenotypes of different anastomosis groups of *Rhizoctonia solani* strains

**DOI:** 10.1186/s12866-024-03363-9

**Published:** 2024-06-20

**Authors:** Meili Sun, Hancheng Wang, Guo Ye, Songbai Zhang, Zhen Li, Liuti Cai, Feng Wang

**Affiliations:** 1 MARA Key Laboratory of Sustainable Crop Production in the Middle Reaches of the Yangtze River (Co-construction by Ministry and Province), Yangtze University, Jingzhou, Hubei, 434025 People’s Republic of China; 2 Guizhou Provincial Academician Workstation of Microbiology and Health, Guizhou Academy of Tobacco Science, Guiyang, 550081 P. R. China

**Keywords:** Tobacco, *Rhizoctonia solani*, Anastomosis group, Metabolic phenotypes

## Abstract

**Background:**

*Rhizoctonia solani* is an important plant pathogen worldwide, and causes serious tobacco target spot in tobacco in the last five years. This research studied the biological characteristics of four different anastomosis groups strains (AG-3, AG-5, AG-6, AG-1-IB) of *R. solani* from tobacco. Using metabolic phenotype technology analyzed the metabolic phenotype differences of these strains.

**Results:**

The results showed that the suitable temperature for mycelial growth of four anastomosis group strains were from 20 to 30^o^C, and for sclerotia formation were from 20 to 25^o^C. Under different lighting conditions, *R. solani* AG-6 strains produced the most sclerotium, followed by *R. solani* AG-3, *R. solani* AG-5 and *R. solani* AG-1-IB. All strains had strong oligotrophic survivability, and can grow on water agar medium without any nitrutions. They exhibited three types of sclerotia distribution form, including dispersed type (*R. solani* AG-5 and AG-6), peripheral type (*R. solani* AG-1-IB), and central type (*R. solani* AG-3). They all presented different pathogenicities in tobacco leaves, with the most virulent was noted by *R. solani* AG-6, followed by *R. solani* AG-5 and AG-1-IB, finally was *R. solani* AG-3. *R. solani* AG-1-IB strains firstly present symptom after inoculation. Metabolic fingerprints of four anastomosis groups were different to each other. *R. solani* AG-3, AG-6, AG-5 and AG-1-IB strains efficiently metabolized 88, 94, 71 and 92 carbon substrates, respectively. Nitrogen substrates of amino acids and peptides were the significant utilization patterns for *R. solani* AG-3. *R. solani* AG-3 and AG-6 showed a large range of adaptabilities and were still able to metabolize substrates in the presence of the osmolytes, including up to 8% sodium lactate. Four anastomosis groups all showed active metabolism in environments with pH values from 4 to 6 and exhibited decarboxylase activities.

**Conclusions:**

The biological characteristics of different anastomosis group strains varies, and there were significant differences in the metabolic phenotype characteristics of different anastomosis group strains towards carbon source, nitrogen source, pH, and osmotic pressure.

**Supplementary Information:**

The online version contains supplementary material available at 10.1186/s12866-024-03363-9.

## Highlights

Phenotyping differences in carbon, nitrogen, and pH metabolism, as well in osmotic pressure among four anastomosis groups (AG-3, AG-5, AG-6, AG-1-IB) of *R. solani* were different.

Nitrogen substrates of amino acids and peptides were the main metabolic substrates for *R. solani* AG-3.

*R. solani* AG-3 and AG-6 showed a wide range of adaptability.

**Supplementary Information:**

The online version contains supplementary material available at 10.1186/s12866-024-03363-9.

## Background

Tobacco (*Nicotiana tabacum* L.) is a major commercial crops in China, with an annual planting area of up to 1 million hectares. China produces nearly 40% of the total global tobacco leaves and 40% of the global tobacco consumption [1]. Tobacco target spot is a devastating disease on tobacco production. It can occur from the seedling stage to the mature stage, while mainly harming tobacco leaves in the field [2]. At the early stage, the disease spots are circular water stains, and the tobacco leaves fade with yellow halos. Laterly, the disease spots expand and form the disease spot with a diameter of 2–3 cm. Typical symptoms of tobacco target spots firstly appear on the old leaves as round watery spots, the tobacco leaves are chlorotic, and with yellow halo [3]. The necrotic parts of the disease spots are fragile, resembling cavities left on the target after gunshot. When humidity rises, the edge of the lesion often produces the mycelium of its pathogen, and future occur fruiting layer and basidiospores of the sexual generation [4]. Then the multiple lesions are connected into patches and leading to perforation and rupture of the leaves, thereby depriving the leaf of its economic value [5].

The pathogen of tobacco target spot is *Rhizoctonia solani* Kühn. *R. solani* has affected more than 260 important cash crops, such as potatoes, tomatoes, sugar, corn, wheat and peanuts [6–9]. In recent years, tobacco target spot has occurred in Guizhou, Chongqing, Yunnan, Sichuan and other major tobacco producing areas [10, 11]. The disease incidence rate of tobacco plants can reach more than 80%, and even more 100% [12], reduce the value of tobacco leaves. *R. solani* belongs to the Hyphomycetes, Agonomycetales, Agonomycetaceae and *Rhizoctonia* [13]. It does not produce conidium, and its sexual generation is *Thanatephorus cucumber* (Frank) Donk [14]. The genetic differentiation of *R. solani* is complex and its life history is relatively unique [13], and a phenomenon commonly occurring in filamentous fungi has been pointed out as hyphal anastomosis, which is characterized by the exchange of genetic material [15]. In a case study, it has been reported that the mycelial anastomosis phenomenon taxa of *R. solani* and established the system of mycelial anastomosis group (anastomosis group, AG for short) [16]. The existence of 14 mycelial anastomosis groups of *R. solani* has been reported, including AG-1 to AG-13 and AG-B1 [17]. Ogoshi further subdivided the anastomosis group into 18 subgroups of *R. solani* based on the anastomosis group [18]. The *R. solani* AG-3, which was first identified and reported to have the widest distribution range on tobacco in China [19]. In another study identified the anastomosis group of tobacco target spot pathogens in some tobacco areas of Hunan Province and found that tobacco target spot pathogens belong to *R. solani* AG-3 [20]. In a recent study identified the anastomosis group of tobacco target spot pathogens in Hubei Province, China, and found that tobacco target spot pathogens belong to *R. solani* AG-3 [21]. Other anastomosing groups of *R. solani* have been reported in most tobacco areas of China. Chen et al. identified the anastomosis group of tobacco target spot pathogens in Guangxi Province and found that the pathogens belong to *R. solani* AG-2 and *R. solani* AG-4 [22]. Our laboratory identified the anastomosis group of *R. solani* in tobacco regions of Guizhou and Sichuan provinces in the early stage, which belongs to *R. solani* AG-5 and *R. solani* AG-6. This is also the first report of tobacco target spot caused by *R. solani* AG-5 and *R. solani* AG-6 on tobacco in China [23, 24].

Biolog metabolic phenotype technology is one of the important methods for studying microbial metabolic function. It is a technology invented by Bochner in the United States in 2000 for measuring cell phenotype [25, 26]. The system can measure nearly 1000 metabolic phenotypes of microorganisms, and can be used in conjunction with computer software for data analysis. It has the characteristics of high automation and standardization, and fast identification speed [27]. It has microporous plates such as GEN III microplates, ECO metabolic plates, FF microplates, and Phenotype Microarray (PM) microplates. Its principle is that during the metabolic process of microbial cells, the free electrons generated by the metabolic carbon / nitrogen substrate undergo a reduction color reaction and turbidity difference with tetrazole dyes [26]. By utilizing a unique phenotypic arrangement technique, the metabolic fingerprint of each microorganism can be detected [28]. This technique can also be used to study the metabolic function of environmental microbial populations. In many studies, it has been used to analyze the activity of microbial communities, or to study the pathogenic mechanism of pathogens and the action mechanism of fungicides through metabolic conditions [28–31]. In tobacco, Wang et al. used ECO metabolic plates to research the differences in metabolic function of tobacco brown spot pathogen [30]. Similarly, Liu et al. used the ECO metabolic plates to study the metabolic function of microorganisms in the rhizosphere of tobacco leaves with different maturity levels susceptible to brown spot disease [32]. In another study, Wang et al. used Biolog FF microplates to determine the biological activity of azoxystrobin, and salicyloximic acid against *Fusarium oxysporum* strain from tobacco [1]. In a recent study, Liu et al. used the PM 9–10 microplates to study the metabolic phenotype on different osmotic pressure and pH environments of tobacco black shank pathogen [33]. Previous researchers have conducted in-depth and systematic studies on tobacco brown spot pathogen, black shank pathogen, and powdery mildew pathogen using this metabolic phenotype technology. Nevertheless, there have no reports on the use of metabolic phenotype technology to study tobacco target spot pathogen, and the biological characteristics of different anastomosis groups strains have not reported.

Therefore, the research measured the biological characteristics of different anastomosis group strains, and measured the metabolic phenotypic characteristics of different anastomosis group mycelial to carbon substrate, nitrogen substrate, pH and osmotic pressure using the Biolog metabolic phenotype technology. Determine the pathogenicity of tobacco target spot pathogens in different anastomosis groups on K326 tobacco leaves. The objective of this research was to (i) identify biological characteristics of four anastomosis groups of *R. solani* and (ii) characterize the metabolic phenotype of four anastomosis groups of *R. solani*. The data provided by this study will be valuable to expanding the knowledge of the biochemistry and metabolic phenomics of *R. solani* strains and will ideally assist in the development of more effective measures for tobacco target spot and tobacco sore shin management.

## Results

### Effect of different temperatures on mycelial growth and sclerotium formation of *R*. *solani* at different anastomosis groups

**Fig. 1:**
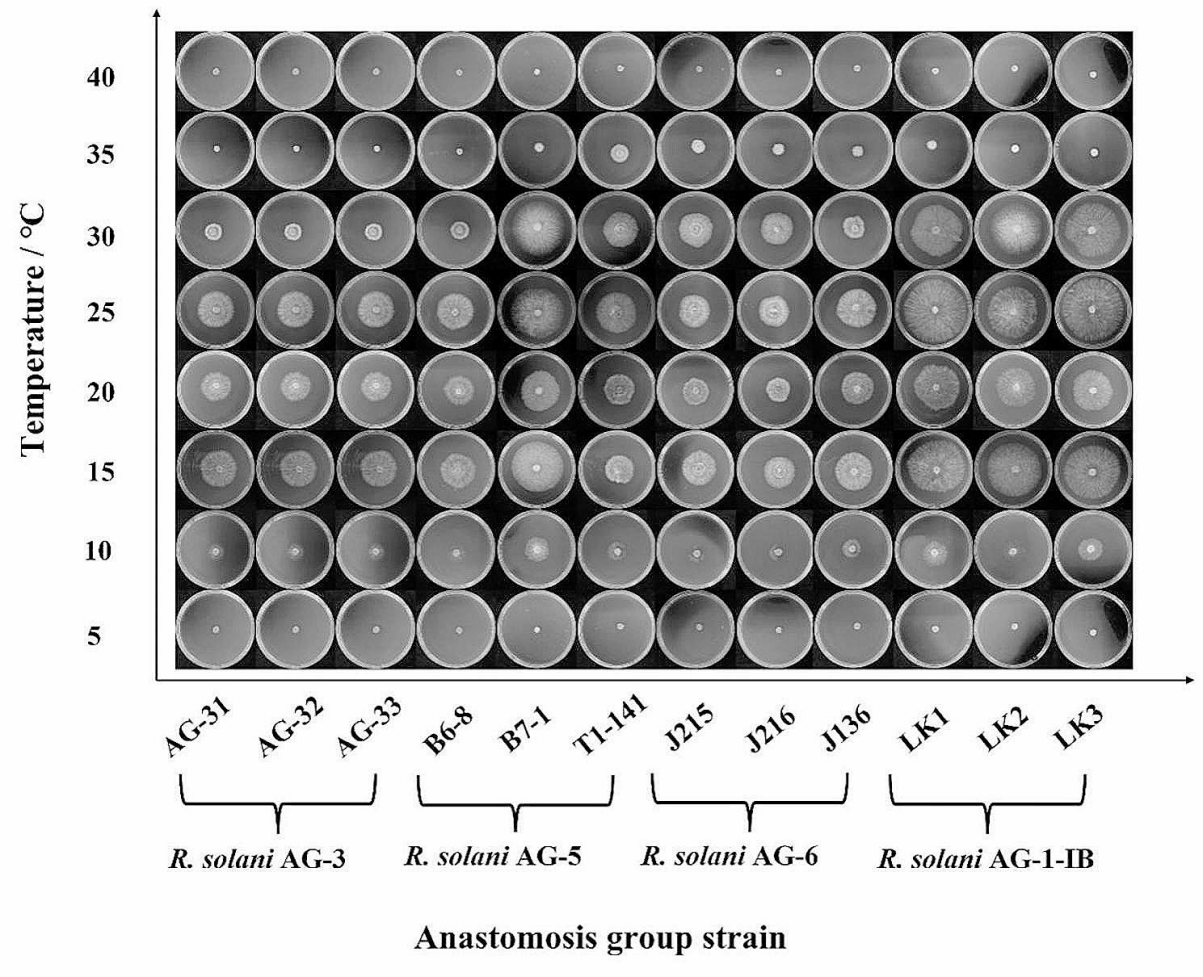
Colony morphology of different anastomosis group strains of *Rhizoctonia solani* at different temperatures

The temperature range is 10℃-35℃ of different anastomosis group strains’ mycelium can grow. The mycelium of four anastomosis group strains can’t grow at too low (5℃) or too high (40℃) temperature, the mycelium grew fastest at 15 and 25℃, followed by 20 and 30℃ (Table 1; Fig. 1). The results of variance analysis showed that there were significant differences between the colony diameters of different anastomosis group strains at the same temperature. The mycelium of *R. solani* AG-1-IB strains grew fastest at 10, 15, 20, 25 and 30 °C compared to other anastomosis group strains and there were significant differences. The mycelium of *R. solani* AG-6 strains grew fastest at 35 °C, followed by *R. solani* AG-5 and *R. solani* AG-1-IB, and finally the *R. solani* AG-3. There were significant differences in colony diameters among the four anastomosis group strains.

**Table 1:** Effects of temperature on mycelium growth of different anastomosis group of *Rhizoctonia solani* strains

Anastomosis groups	Strains	Temperature / ^o^C								
			5	10	15	20	25	30	35	40
*R. solani* AG-3	AG-31		6.00 ± 0.00a	21.50 ± 1.38c	40.83 ± 0.75 cd	36.00 ± 1.90bc	42.17 ± 3.37de	19.50 ± 0.84e	7.00 ± 0.00c	6.00 ± 0.00a
	AG-32		6.00 ± 0.00a	21.50 ± 1.38c	40.67 ± 2.25 cd	35.67 ± 1.97bc	42.17 ± 3.37de	19.67 ± 1.21e	7.00 ± 0.00c	6.00 ± 0.00a
	AG-33		6.00 ± 0.00a	21.50 ± 1.38c	40.83 ± 1.17 cd	36.17 ± 3.06bc	42.17 ± 3.71de	20.17 ± 0.98e	7.00 ± 0.00c	6.00 ± 0.00a
*R. solani* AG-5	B6-8		6.00 ± 0.00a	22.17 ± 1.17bc	40.50 ± 1.05 cd	36.00 ± 1.41bc	42.33 ± 1.37de	22.50 ± 1.52de	10.83 ± 0.75b	6.00 ± 0.00a
	B7-1		6.00 ± 0.00a	26.50 ± 1.38a	62.17 ± 3.06a	50.50 ± 4.28a	60.83 ± 1.17b	62.33 ± 1.03a	11.00 ± 0.63b	6.00 ± 0.00a
	T1-141		6.00 ± 0.00a	19.67 ± 4.18 cd	30.33 ± 3.88e	35.83 ± 1.17bc	47.50 ± 1.76d	38.33 ± 2.25c	10.67 ± 0.52b	6.00 ± 0.00a
*R. solani* AG-6	J215		6.00 ± 0.00a	17.17 ± 2.93d	38.67 ± 1.37 cd	31.00 ± 1.26 cd	38.17 ± 2.86ef	39.00 ± 0.63c	13.33 ± 2.73a	6.00 ± 0.00a
	J216		6.00 ± 0.00a	16.33 ± 2.80d	35.00 ± 2.10de	28.83 ± 0.75d	35.83 ± 0.98f	38.50 ± 1.76c	13.00 ± 0.89a	6.00 ± 0.00a
	J136		6.00 ± 0.00a	21.50 ± 1.52c	42.67 ± 1.21c	38.33 ± 1.21b	44.83 ± 1.33d	27.83 ± 3.71d	13.17 ± 1.47a	6.00 ± 0.00a
*R. solani* AG-1-IB	LK1		6.00 ± 0.00a	26.50 ± 1.22a	67.00 ± 5.97a	49.00 ± 5.51a	73.33 ± 2.66a	60.33 ± 2.07a	9.33 ± 0.52b	6.00 ± 0.00a
	LK2		6.00 ± 0.00a	26.00 ± 1.10ab	54.17 ± 6.46b	46.00 ± 1.26a	55.17 ± 4.54c	50.50 ± 6.69b	9.33 ± 0.52b	6.00 ± 0.00a
	LK3		6.00 ± 0.00a	26.33 ± 0.82a	61.33 ± 3.39a	46.17 ± 4.79a	70.83 ± 2.93a	63.00 ± 6.93a	9.17 ± 0.41b	6.00 ± 0.00a

Note Different lowercase letters in the same column represented significant difference (*P* < 0.05), the same as below

The sclerotium production of different anastomosis group strains was affected by different temperatures, and both low and high temperatures could affect the sclerotium production. Four anastomosis group strains can’t produce sclerotium at 5, 10, 30, 35 and 40 °C, but the *R. solani* AG-1-IB anastomosis group produced sclerotium after 240 h of cultivation at 15 °C (Table 2). There was no significant difference between the numbers of sclerotium by the three strains of *R. solani* AG-1-IB. All strains produced sclerotium at 20 °C. The first one produced sclerotium was *R. solani* AG-1-IB, followed by *R. solani* AG-3 and *R. solani* AG-5, and finally was *R. solani* AG-6, and the sclerotium formation time were 216 h, 264 h, 408 h, 456 h. There were significant differences between the numbers of sclerotium by different anastomosis group strains. All strains produced sclerotium at 25℃, and the first to produce sclerotium was *R. solani* AG-1-IB, followed by *R. solani* AG-3 and *R. solani* AG-5, and finally was *R. solani* AG-6, and the sclerotium formation time was 168 h, 216 h, 360 h and 408 h. There were significant differences in the number of sclerotium produced by *R. solani* AG-5 and *R. solani* AG-6 strains at 20℃. There were significant differences in the number of sclerotium produced by *R. solani* AG-1-IB and *R. solani* AG-5, *R. solani* AG-6 strains at 25℃. However, there was no significant difference in the number of sclerotium produced by *R. solani* AG-1-IB and *R. solani* AG-3 strains at 25℃ (Table 2).

**Table 2:** Effects of temperature on sclerotium formation of different anastomosis group of *Rhizoctonia solani* strains

Anastomosis groups		Strains		Temperature / ^o^C							
				5	10	15	20	25	30	35	40
Time / h	*R. solani* AG-3		AG-31	-	-	-	264	216	-	-	-
			AG-32	-	-	-	264	216	-	-	-
			AG-33	-	-	-	264	216	-	-	-
	*R. solani* AG-5		B6-8	-	-	-	408	360	-	-	-
			B7-1	-	-	-	408	360	-	-	-
			T1-141	-	-	-	408	360	-	-	-
	*R. solani* AG-6		J215	-	-	-	456	408	-	-	-
			J216	-	-	-	456	408	-	-	-
			J136	-	-	-	456	408	-	-	-
	*R. solani* AG-1-IB		LK1	-	-	240	216	168	-	-	-
			LK2	-	-	240	216	168	-	-	-
			LK3	-	-	240	216	168	-	-	-
Number / per plate	*R. solani* AG-3		AG-31	0	0	0	9.33 ± 1.53bc	40.50 ± 1.73ab	0	0	0
			AG-32	0	0	0	7.67 ± 1.53 cd	40.50 ± 1.73ab	0	0	0
			AG-33	0	0	0	9.00 ± 1.00bcd	40.50 ± 1.73ab	0	0	0
	*R. solani* AG-5		B6-8	0	0	0	6.33 ± 1.53 cd	25.50 ± 8.43b	0	0	0
			B7-1	0	0	0	7.67 ± 0.58 cd	23.50 ± 8.66b	0	0	0
			T1-141	0	0	0	6.00 ± 1.00d	31.75 ± 11.35b	0	0	0
	*R. solani* AG-6		J215	0	0	0	12.00 ± 1.00ab	24.00 ± 5.48b	0	0	0
			J216	0	0	0	13.00 ± 1.00a	24.25 ± 2.63b	0	0	0
			J136	0	0	0	12.00 ± 1.00ab	26.00 ± 2.94b	0	0	0
	*R. solani* AG-1-IB		LK1	0	0	2.33 ± 0.58a	9.00 ± 1.00bcd	56.25 ± 29.44a	0	0	0
			LK2	0	0	2.67 ± 0.58a	7.67 ± 0.58 cd	53.50 ± 27.74a	0	0	0
			LK3	0	0	2.33 ± 0.58a	11.00 ± 1.00ab	61.00 ± 12.78a	0	0	0

*Note* In the table, “-” means that the strains did not form a sclerotium during the observation period

### Effects of different light times on the mycelial growth and sclerotium formation of *R. solani* at different anastomosis groups

**Fig. 2:**
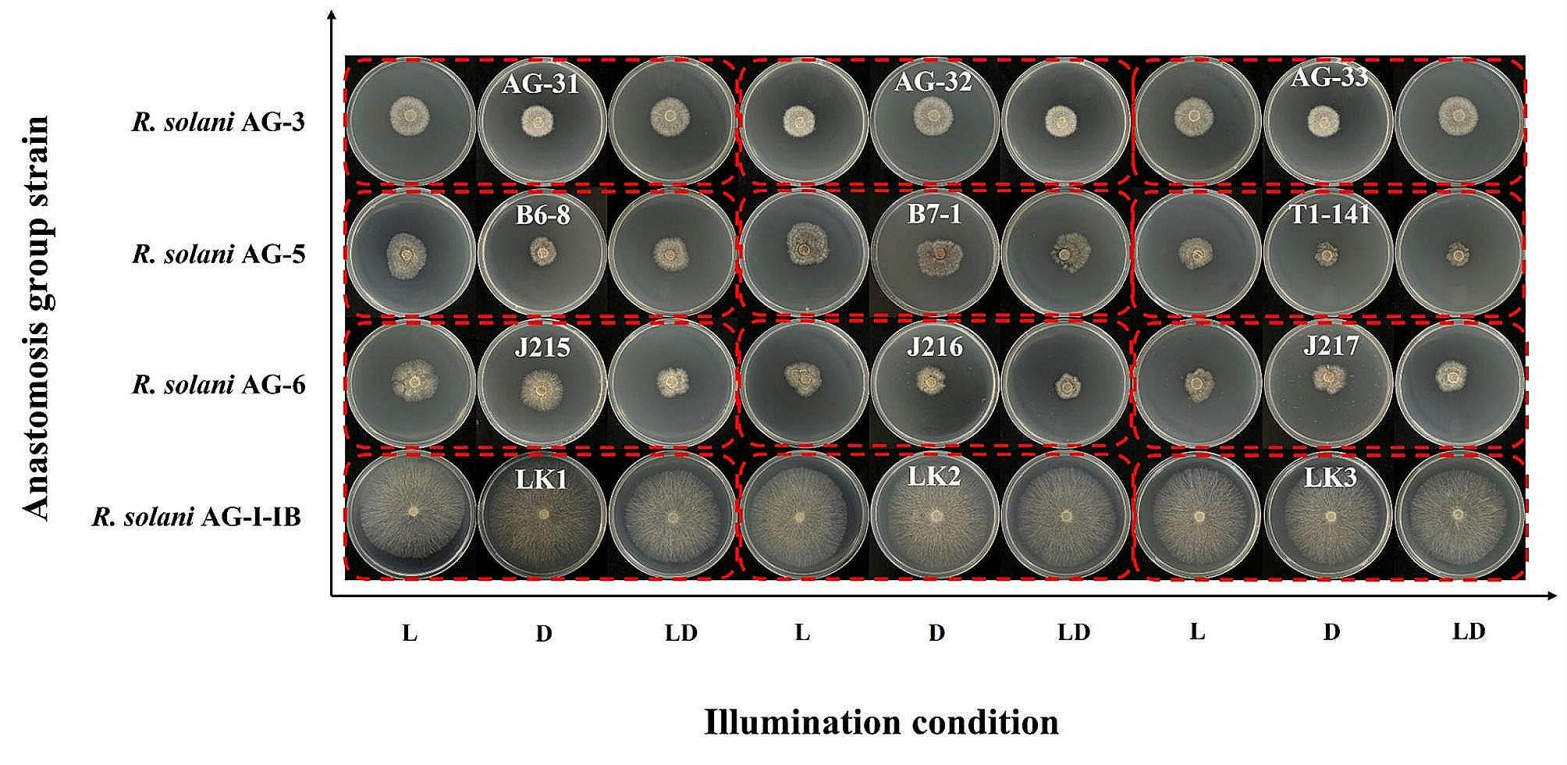
Colony morphology of different anastomosis group strains of *Rhizoctonia solani* under different illumination conditions. *Note* L represents continuous illumination, D represents total darkness, LD represents 12 h of alternating light and dark conditions

The *R. solani* AG-3 strains grew fastest under 12 h of alternating light and dark conditions, followed by total darkness conditions, and finally was continuous illumination conditions (Fig. 2). The mycelial growth of *R. solani* AG-5 strains and the *R. solani* AG-3 was exactly the opposite, with continuous illumination being the fastest condition for mycelium growth, followed by 12 h of alternating light and dark, and finally was total darkness. The fastest growth condition for the mycelium of *R. solani* AG-6 and *R. solani* AG-1-IB was continuous illumination, followed by total darkness, and finally was 12 h of alternating light and dark. There were significant differences in the mycelial growth of different anastomosis group strains under the same lighting conditions. Under continuous illumination conditions, the mycelial growth rates of *R. solani* AG-3 strains were the slowest. There were significant differences in the mycelium growth rates between *R. solani* AG-1-IB strains and *R. solani* AG-3, *R. solani* AG-5, *R. solani* AG-6 strains under continuous illumination. Under total darkness conditions and 12 h of alternating light and dark conditions, there was significant difference in the mycelial growth rate between *R. solani* AG-1-IB strains and *R. solani* AG-3, *R. solani* AG-5, *R. solani* AG-6 strains (Table 3).

The time required for sclerotium formation of different anastomosis group strains varies under different lighting conditions (Table 3). Under continuous illumination conditions, the *R. solani* AG-1-IB strains first produced sclerotium, followed by *R. solani* AG-6 strains, *R. solani* AG-5 strains, and finally was *R. solani* AG-3 strains. The four anastomosis group strains required 96 h, 192 h, 312 h, and 408 h to form sclerotia, respectively. There was no significant difference in the number of sclerotia produced by the four anastomosis group strains under continuous illumination conditions. Under total darkness conditions, the *R. solani* AG-1-IB strains first produced sclerotium, followed by *R. solani* AG-6 strains, *R. solani* AG-5 strains, and finally was the *R. solani* AG-3 atrains. The four anastomosis group strains required 144 h, 216 h, 312 h, and 432 h to form sclerotia, respectively. There were significant differences in the number of sclerotium produced by the four anastomosis group strains under total darkness conditions, and there were significant differences in the number of sclerotium among different strains of the same anastomosis group.

**Table 3:** Effects of illumination on mycelium growth and sclerotium formation of different anastomosis group of *Rhizoctonia solani* strains

Anastomosis groups		Strains	Continuous illumination	Total darkness	12 h of alternating light and dark conditions
Diameter /mm	*R. solani* AG-3	AG-31	22.63 ± 1.11 g	24 ± 1.47 cd	27.00 ± 1.23b
		AG-32	22.63 ± 1.11 g	24 ± 1.47 cd	27.00 ± 1.23b
		AG-33	22.63 ± 1.11 g	24 ± 1.47 cd	27.00 ± 1.23b
	*R. solan* AG-5	B6-8	38.63 ± 2.75e	19.25 ± 1.04ef	22.63 ± 4.42c
		B7-1	33.75 ± 0.87f	25.13 ± 5.04 cd	26.5 ± 3.14b
		T1-141	45.13 ± 2.25c	22.13 ± 4.27de	14.38 ± 1.49e
	*R. solan* AG-6	J215	43.63 ± 1.84 cd	26.50 ± 1.58c	20.75 ± 2.60 cd
		J216	41.25 ± 1.44de	18.50 ± 1.68ef	17.63 ± 1.38de
		J136	43.00 ± 0.41 cd	17.75 ± 2.10f	18.13 ± 3.20de
	*R. solan* AG-1-IB	LK1	72.75 ± 1.85a	73.50 ± 1.78a	62.25 ± 3.57a
		LK2	68.38 ± 3.42b	71.63 ± 3.57a	65.75 ± 1.85a
		LK3	73.13 ± 2.50a	65.13 ± 2.10b	62.25 ± 1.85a
Time / h	*R. solan* AG-3	AG-31	408	432	336
		AG-32	408	432	336
		AG-33	408	360	168
	*R. solan* AG-5	B6-8	384	360	360
		B7-1	360	360	312
		T1-141	312	312	336
	*R. solan* AG-6	J215	216	216	312
		J216	192	216	144
		J136	216	216	120
	*R. solan* AG-1-IB	LK1	96	144	216
		LK2	216	240	144
		LK3	216	216	216
Number / per plate	*R. solan* AG-3	AG-31	55.00 ± 1.41a	40.50 ± 1.73ab	31.00 ± 12.68abc
		AG-32	34.00 ± 1.41ab	25.50 ± 8.43b	32.00 ± 10.55abc
		AG-33	33.50 ± 0.71ab	23.50 ± 8.66b	38.00 ± 13.69abc
	*R. solan* AG-5	B6-8	42.00 ± 2.83ab	31.75 ± 11.35b	33.00 ± 2.16abc
		B7-1	57.00 ± 33.94a	24.00 ± 5.48b	33.67 ± 9.07abc
		T1-141	26.00 ± 8.49b	24.25 ± 2.63b	30.00 ± 2.83bc
	*R. solan* AG-6	J215	44.50 ± 16.26ab	26.00 ± 2.94b	27.25 ± 3.86c
		J216	35.00 ± 7.07ab	56.25 ± 29.44a	45.25 ± 21.84ab
		J136	41.00 ± 0.00ab	53.50 ± 27.74a	45.25 ± 11.59ab
	*R. solan* AG-1-IB	LK1	41.50 ± 9.19ab	61.00 ± 12.78a	47.50 ± 10.63a
		LK2	55.00 ± 1.41a	40.50 ± 1.73ab	31.00 ± 12.68abc
		LK3	34.00 ± 1.41ab	25.50 ± 8.43b	32.00 ± 10.55abc

### Effect of oligotrophic medium on the mycelial growth and sclerotium formation of *R. solani* at different anastomosis groups

**Fig. 3:**
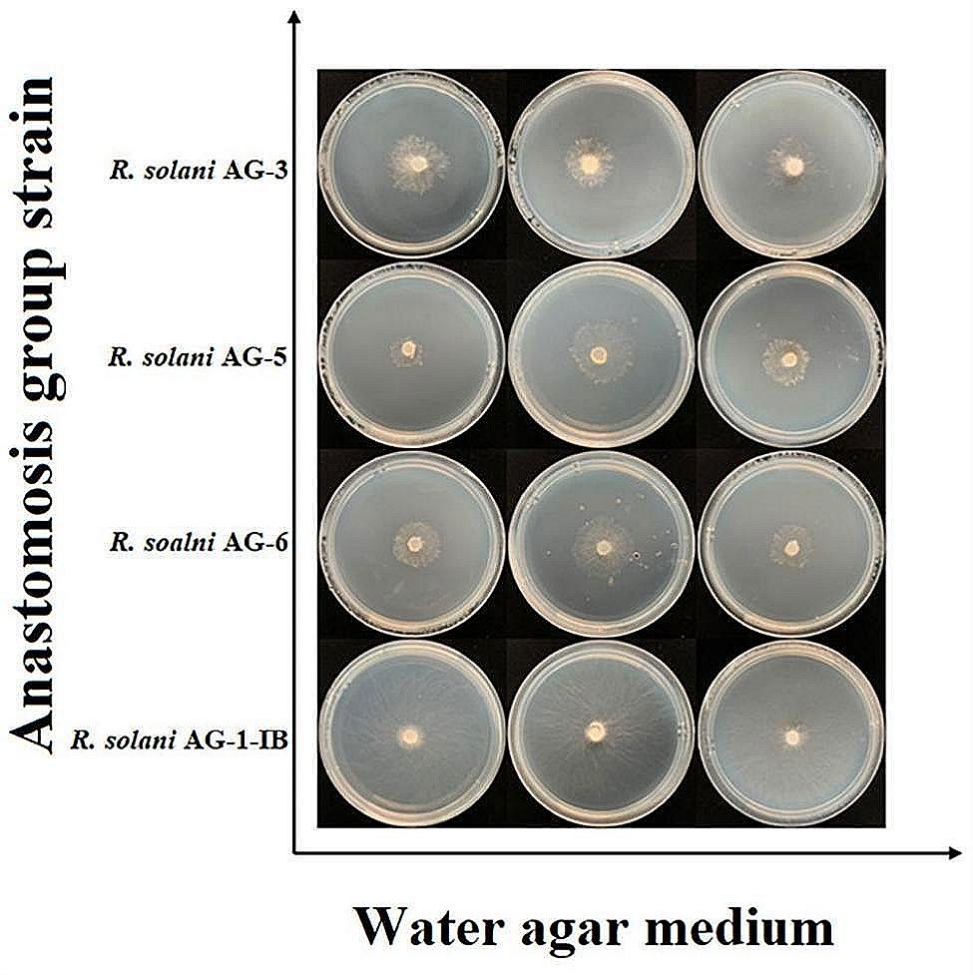
Colony morphology of different anastomosis group strains of *Rhizoctonia solani* on water agar medium

The mycelium of different anastomosis group strains can grow on oligotrophic medium (water agar medium), but the growth rate varies, and the *R. solani* AG-1-IB strains had the fastest growth rate (Fig. 3; Table 4). The significant differences were observed between colony diameters of strains of different anastomosis groups, while significant differences were observed between strains AG-31 and strains AG-32, strains AG-33 of *R. solani* AG-3, and between colony diameters of three strains of *R. solani* AG-5 and *R. solani* AG-6. The oligotrophic medium inhibited the formation of sclerotium in the different anastomosis groups, and all strains of *R. solani* AG-3, *R. solani* AG-5 and *R. solani* AG-6 can’t form sclerotium during the 50 d observation period, while only the *R. solani* AG-1-IB strains formed sclerotium, but the number of sclerotium was very small (Fig. 3; Table 4).

**Table 4:** Effect of water agar medium on mycelial growth and sclerotia formation of different anastomosis group of *Rhizoctonia solani* strains

Anastomosis group	Strain	Diameter / mm	Time / h	Number / per plate
*R. solan* AG-3	AG-31	36.33 ± 1.04b	-	0
	AG-32	31.50 ± 1.00c		0
	AG-33	32.33 ± 4.73c		0
*R. solan* AG-5	B6-8	18.25 ± 0.75f	-	0
	B7-1	36.00 ± 0.50b		0
	T1-141	28.00 ± 0.50d		0
*R. solan* AG-6	J215	28.25 ± 0.25d	-	0
	J216	32.25 ± 3.75c		0
	J136	23.50 ± 1.00e		0
*R. solan* AG-1-IB	LK1	80.25 ± 0.25a	504 h	6
	LK2	80.50 ± 0.50a		7
	LK3	82.00 ± 0.00a		8

*Note* In the table, “-” means that the strains did not form a sclerotium during the observation period

### Analysis of differences in sclerotium formation of *R. solani* at different anastomosis groups

**Fig. 4:**
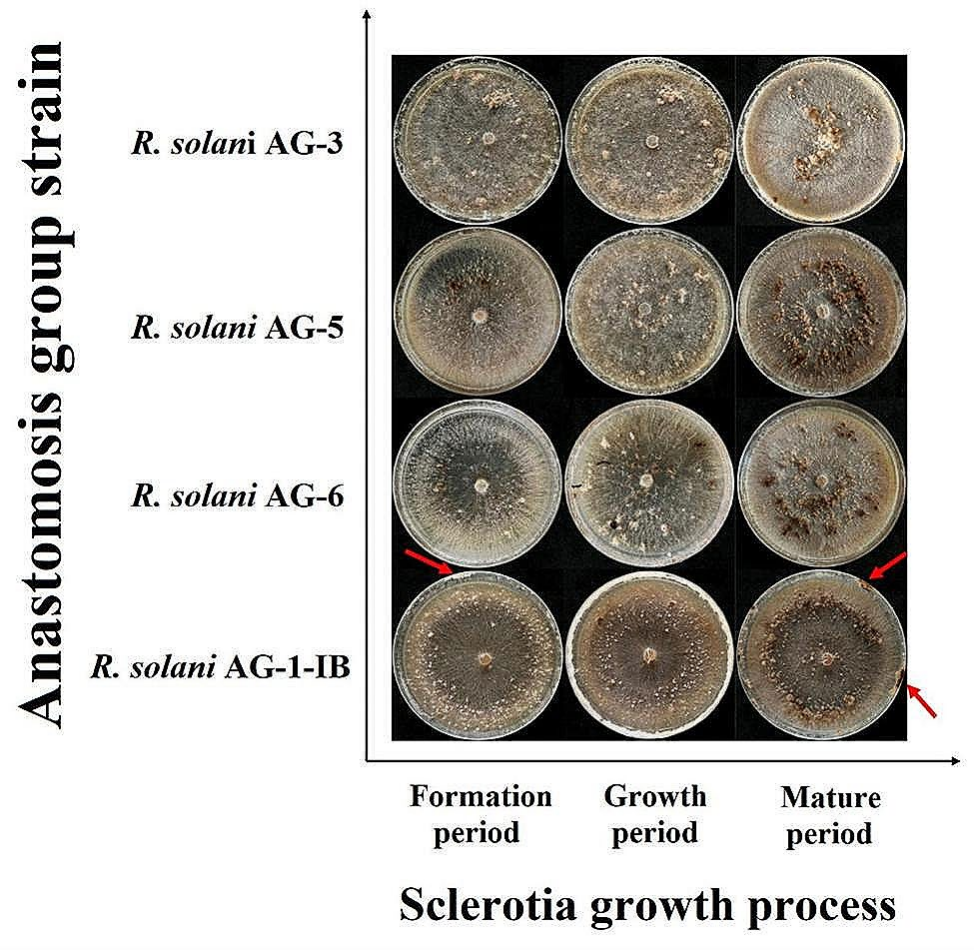
Sclerotium morphology of different anastomosis group strains of *Rhizoctonia solani. Note* The red arrow in the figure represents the mycelium growing along the edge of the culture dish and eventually forming sclerotium

The four anastomosis group strains can be divided into three types based on the distribution form of sclerotia, including dispersed type, peripheral type, and central type (Fig. 4). The sclerotium distribution pattern of *R. solani* AG-3 was central type. The sclerotium distribution pattern of *R. solani* AG-5 and *R. solani* AG-6 were dispersed type. The sclerotium distribution pattern of *R. solani* AG-1-IB was peripheral type, and during the sclerotia formation stage, it is observed that some mycelial will grow along the edge of the culture dish and eventually form sclerotia, as shown by the red arrow in Fig. 4. During the observation period, the *R. solani* AG-1-IB strains had the highest number of sclerotium, followed by *R. solani* AG-3 strains, finally was the *R. solani* AG-5 strains and *R. solani* AG-6 strains (Table 5). The *R. solani* AG-1-IB strains required the shortest time for sclerotium formation, followed by *R. solani* AG-3 strains and *R. solani* AG-6 strains, finally was the *R. solani* AG-5 strains. In terms of the sclerotium number, the *R. solani* AG-1-IB strains can form the most sclerotium. There were significant differences in the number of sclerotium between the *R. solani* AG-1-IB strains and the *R. solani* AG-5 strains, the *R. solani* AG-6 strains. However, the number of sclerotium formed by *R. solani* AG-1-IB strains was higher than that the *R. solani* AG-3 strains, and there was no significant difference at the sclerotium number between *R. solani* AG-1-IB strains and *R. solani* AG-3 strains. There was no significant difference in the sclerotium number between the *R. solani* AG-5 and the *R. solani* AG-6 strains (Table 5).

**Table 5:** Time and quantity of sclerotia formation of different anastomosis group of *Rhizoctonia solani* strains

Anastomosis group	Strain	Time / h	Number / per plate
*R. solan* AG-3	AG-31	216	40.50 ± 1.73ab
	AG-32		40.50 ± 1.73ab
	AG-33		40.50 ± 1.73ab
*R. solan* AG-5	B6-8	408	25.50 ± 8.43b
	B7-1		23.50 ± 8.66b
	T1-141		31.75 ± 11.35b
*R. solan* AG-6	J215	360	24.00 ± 5.48b
	J216		24.25 ± 2.63b
	J136		26.00 ± 2.94b
*R. solan* AG-1-IB	LK1	168	56.25 ± 29.44a
	LK2		53.50 ± 27.74a
	LK3		61.00 ± 12.78a

### Differences in pathogenicity of *R. solani* at different anastomosis groups on K326 tobacco leaf

**Fig. 5:**
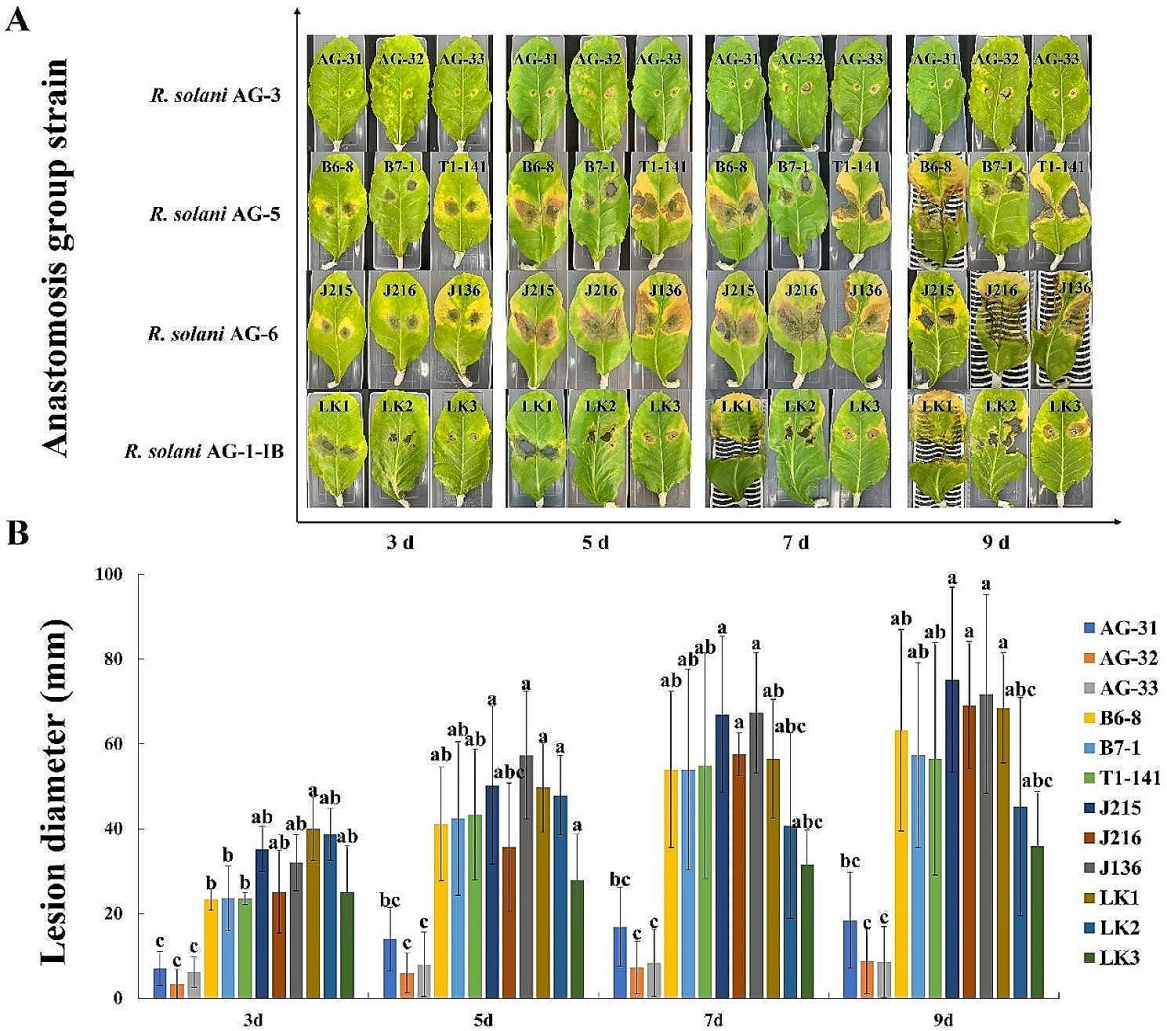
Pathogenicity of different anastomosis groups of *Rhizoctonia solani* on K326 tobacco

Daily observations revealed the leaves inoculated with the *R. solani* AG-1-IB strains were the first to develop lesion, followed by *R. solani* AG-5 strains and *R. solani* AG-6 strains, and finally was *R. solani* AG-3 strains. The largest lesion diameter at 9 d of disease lesion was on leaves inoculated with the *R. solani* AG-6 strains, followed by leaves inoculated with the *R. solani* AG-5 strains and leaves inoculated with the *R. solani* AG-1-IB strains, and finally was leaves inoculated with the *R. solani* AG-3 strains (Fig. 5). The *R. solani* AG-3 strains colonized on leaves and exhibited the light symptoms. The *R. solani* AG-5, *R. solani* AG-6 and *R. solani* AG-1-IB strains colonized on leaves and exhibited extremely severe symptoms. On the 9th day of disease lesion, the diseased leaves of each anastomosis group strains showed severe damage (Fig. 5A). The pathogenicity of the *R. solani* AG-5, *R. solani* AG-6, and *R. solani* AG-1-IB strains was significantly different from that of the *R. solani* AG-3 strains at 3 d of disease lesion. The pathogenicity of the *R. solani* AG-6 strains was significantly different from that of the *R. solani* AG-3 strains at 3, 7 and 9 d of disease lesion (Fig. 5B). In summary, the pathogenicity of different anastomosis group strains differed on the main tobacco varieties. The *R. solani* AG-1-IB strains being the first to colonize the leaves and show symptoms, and the *R. solani* AG-6 strains being the severe pathogenic (Fig. 5).

### Phenotype differences in carbon substrate metabolism among different anastomosis groups of *R*. *solani*

**Fig. 6:**
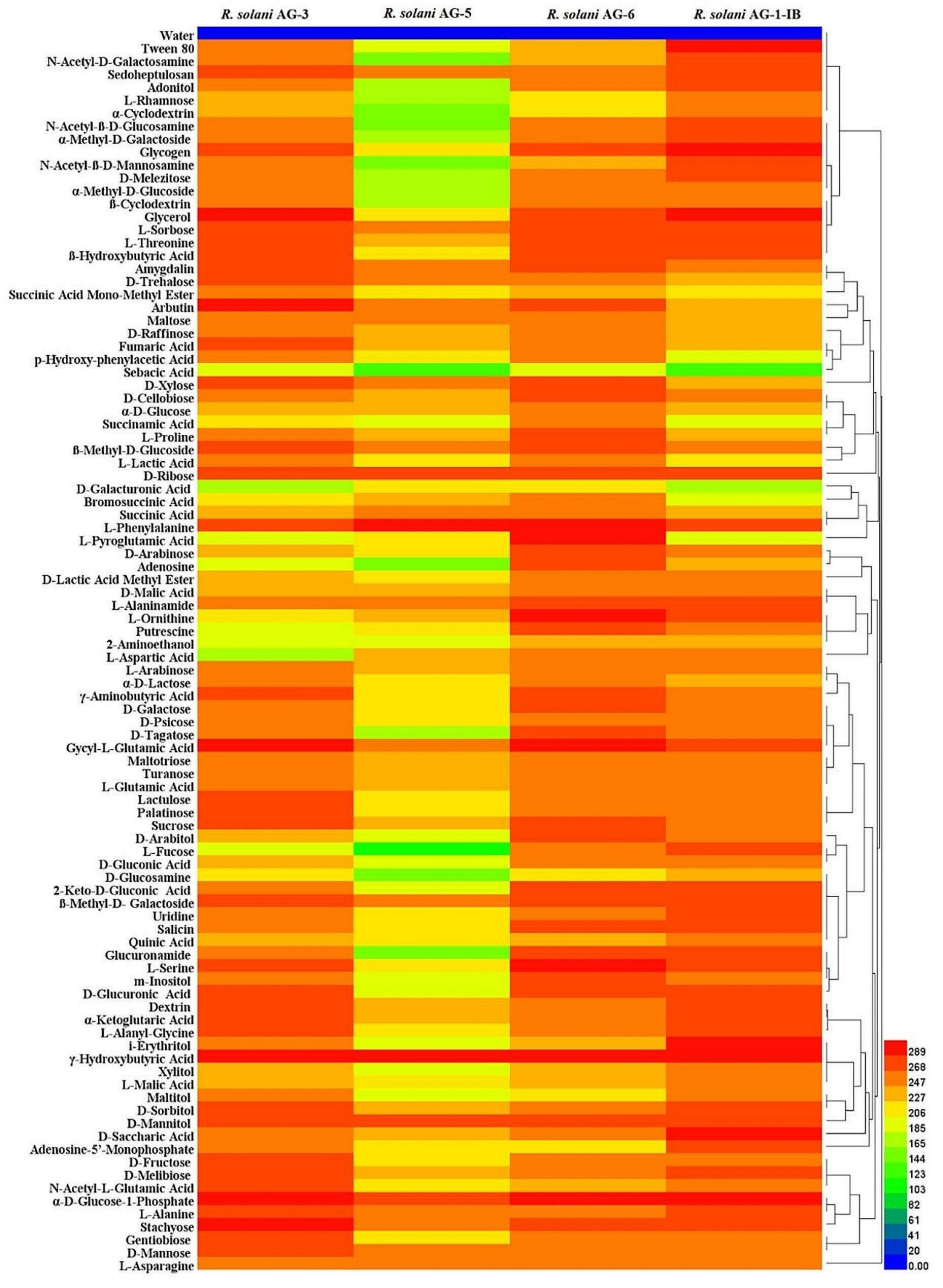
Heat map of 95 carbon sources metabolism abundance of different anastomosis group strains of *Rhizoctonia solani Note* The legend of colour code from blue to green, and red shades indicate low, moderate, and high utilization of carbon sources, respectively, assessed as arbitrary Omnilog values

The research found that the strains from different anastomosis group can metabolize all carbon substrates in the FF microplates. The *R. solani* AG-1-IB strains had a strong metabolic ability towards other carbon substrates, in addition to a weaker metabolic ability towards D-Galacturonic Acid and Sebacic Acid. The *R. solani* AG-6 strains can metabolize 95 carbon substrates and had a strong ability to metabolize carbon substrates. The *R. solani* AG-5 strains had a strongest ability to metabolize α-D-Glucose-1-Phosphate, D-Mannitol, γ-Hydroxybutyric Acid, L-Phenylalanine, while had a weaker ability to metabolize other 91 carbons. The *R. solani* AG-3 strains can metabolize 95 carbon substrates, among which the utilization ability of D-Galactiuronic Acid, L-Aspartic Acid, L-Fucose, *D*-Glucosamine, Bromosuccinic Acid, Sebacic Acid, L-Pyroglutamic Acid, 2-Aminoethanol, Putrescine, Adenosine was weak (Fig. 6).

### Phenotype differences in nitrogen substrate metabolism among different anastomosis groups of *R*. *solani*

**Fig. 7:**
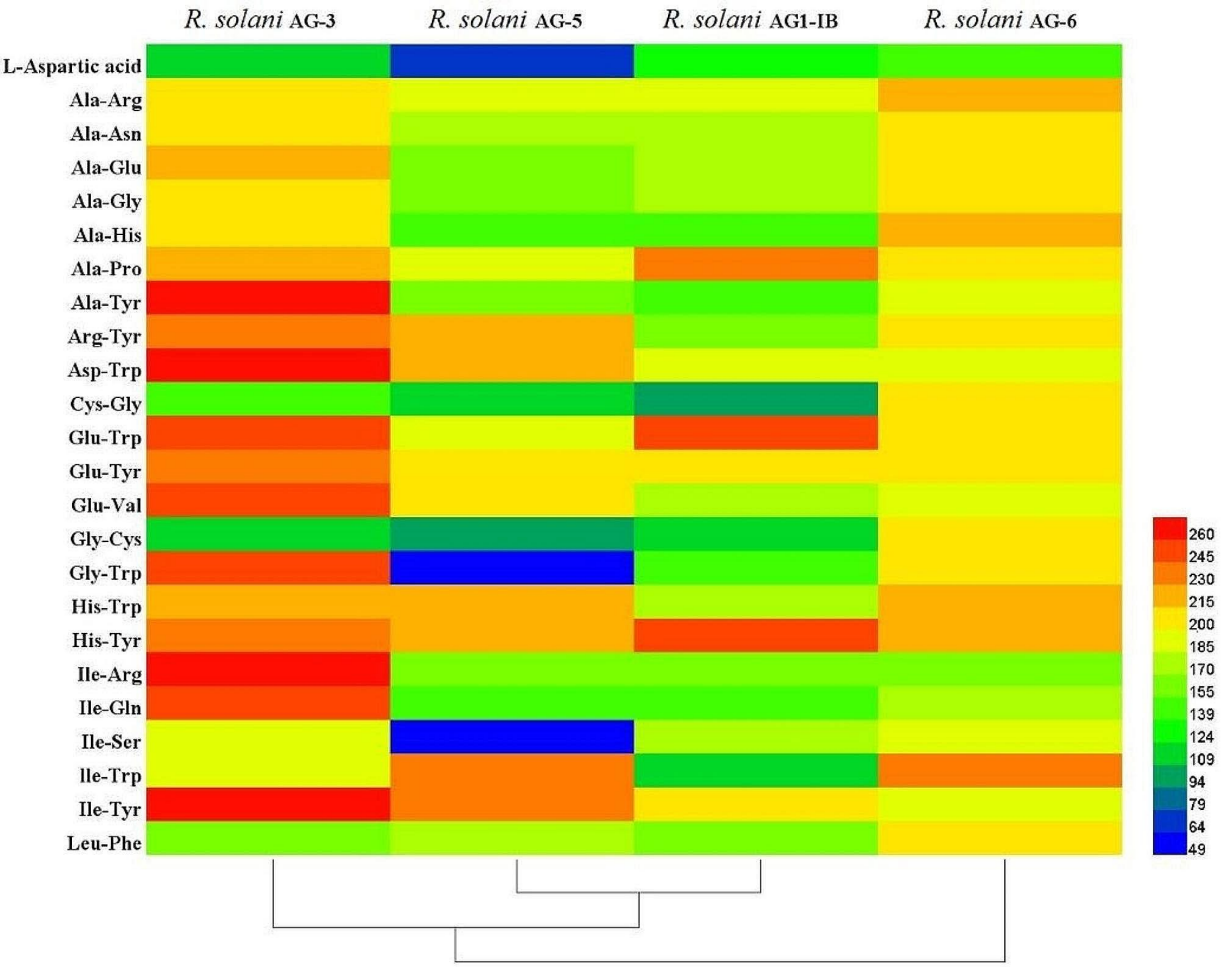
Heat map nitrogen sources metabolism abundance of different anastomosis group strains of *Rhizoctonia solani*

The research found that the strains from different anastomosis group can metabolize all nitrogen substrates in the PM 5 and PM 6 microplates with different metabolic capacities. Nitrogen substrates that were efficiently metabolized by the *R. solani* AG-3 strains include Ile-Arg, Ile-Tyr, Glu-Trp, Glu-Val, Asp-Trp, IIe-Gln and Ala-Tyr. The *R. solani* AG-5 strains can efficiently metabolize a few substrates such as lle-Trp and IIe-Tyr, and unable to metabolize L-Aspartic acid, Ile-Ser, Gly-Trp. The *R. solani* AG-6 strains was able to metabolize all nitrogens and can be metabolized efficiently include lle-Trp, His-Trp, Ala-His, His-Tyr, Ala-Arg, Cys-Gly, Ala-Pro, Ala-Asn, Leu-Phe, Gly-Trp, Ala-Gly, Arg-Tyr, Glu-Tyr, Gly-Cys, Ala-Glu, Glu-Trp, while the other substrates have low metabolizing ability. The *R. solani* AG-1-IB strains can efficiently metabolize His-Tyr, Glu-Trp, Ala-Pro, Ile-Tyr and Glu-Tyr, and can metabolize other substrates with low metabolic rate except for lle-Phe (Fig. 7, Figure S1).

### Phenotype differences in osmotic pressure and Ph metabolism among different anastomosis groups of *R*. *solani*

**Fig. 8:**
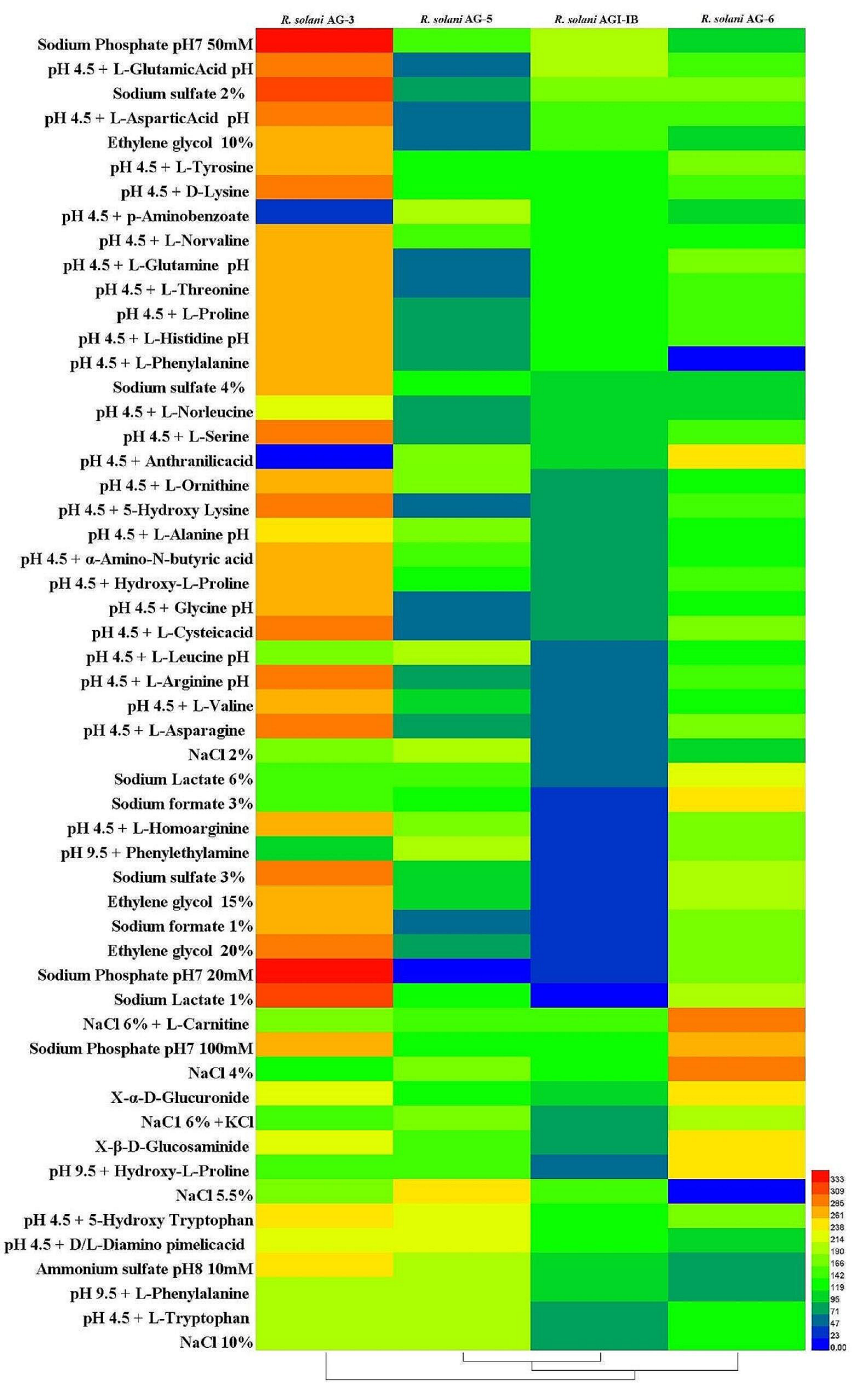
Heat map of pH and osmotic pressure metabolism abundance of different anastomosis group strains of *Rhizoctonia solani*

The strains of four anastomosis groups had weak adaptability to Osmotic pressure and pH environment, among which *R. solani* AG-3 strains has the most extensive adaptability to pH. At pH = 4.5, it can grow in an environment where 22 substances, including L-Alanine, L-Arginine, L-Asparagine, and L-Aspartic Acid, coexist respectively. It can grow in the environment of Sodium sulfate 2%, Sodium sulfate 3%, Sodium sulfate 4%, Ethylene glycol 10%, Ethylene glycol 15%, Ethylene glycol 20%. The *R. solani* AG-5 strains can only grow under four kinds of Osmotic pressure, namely, Social formate 1%, Social formate 2%, Social Lactate 1%, and Social Nickel 10mM. It can’t grow in five kinds of environments where pH and nutrients coexist, namely, pH 4.5 + L-Leucine, pH 4.5 + Anthranilicacid, pH 4.5 + L-Norleucine, pH 4.5 + p-Aminobenzoate, pH 9.5 + Phythylamine. The *R. solani* AG-6 can grow under 21 kinds of Osmotic pressure, including NaCl 2%, Sodium formate 1%, Sodium formate 2%, and Sodium Lactate 1%, but cannot grow under pH 4.5 + Anthranilicacid. The *R. solani* AG-1-IB can grow in all pH or pH coexisting environments with nutrients, but has a low metabolic rate of nutrients (Fig. 8, Figure S2).

## Discussion

The *Rhizoctonia solani* is a destructive fungal pathogen distributed worldwide. Many molecular biological, genetic and genomic studies have been conducted on *R. solani* [34, 35]. Although this pathogen is commonly found in tobacco, potato, rice, wheat and cucumber hosts, the biological characteristics and metabolic phenotypic diversity of *R. solani* and its different anastomosis groups is still poorly understood [36]. The Biolog FF system and Biolog PM system have received considerable attention in population studies of many microorganisms [37]. This study not only studied the biological characteristics of four anastomosis groups of *R. solani* strains, but also the metabolic ability of four anastomosis groups of *R. solani* strains obtained from tobacco was systematically studied using Biolog FF microplates, Biolog PM microplates and important metabolic diversity information was obtained. The data obtained in this study on *R. solani* and its optimal growth conditions, metabolic functional diversity, pathogenic differences can play a very significant role in developing prevention technologies for leaf spot caused by *R. solani* on tobacco.

The *R. solani* can infect more than 200 plant species worldwide [38, 39]. Different anastomosis groups can infect different crops, such as *R. solani* AG-3, which mainly infects tobacco and potato [40–44]. At the same time, in a recent study revealed that *R. solani* AG-5 and *R. solani* AG-6 can infect tobacco [24, 25]. From previous work, it has been demonstrated that different crops have different nutrition substrates, different osmolytes and pH environments in their tissues, which affect the survival and pathogenicity of pathogens [45, 46]. The hosts of *R. solani* are differ in their taxonomy, and the biological characteristics metabolic phenotypic characterization of *R. solani* strains also differ greatly.

Temperature and light have a significant effect on the biological characteristics of *R. solani*. The results of this study showed that the four *R. solani* anastomosis groups stranis can grow at 10 –35 °C, and 20 –25 °C was suitable for the growth of the sclerotium, which was basically consistent with the result of Wu et al. [14]. When the temperature exceeded 25℃, the mycelial of *R. solani* growth was slow, and when the temperature exceeded 35℃, the mycelial of *R. solani* growth stop. Based on this result, temperature control measures can be taken in tobacco seedlings to prevent mycelium of *R. solani* from colonizing the seedlings and to reduce seedling diseases. This result can provide more measures for the prevention and control of tobacco target spot. For example, in tobacco production, when the soil temperature reaches 25℃, it can be used as the first application time to prevent and control tobacco target spot. At the same time, before and after the tobacco plant enters the bud stage, the soil temperature can be reduced by opening the plastic film to reduce the occurrence of the disease. Illumination has an impact on the mycelial growth and sclerotium formation of *R. solani*. This study found that under the condition of 12 h of alternating light and dark, the *R. solani* AG-3 strains had the fastest mycelial growth. Under continuous illumination conditions, the *R. solani* AG-6 strains and *R. solani* AG-1-IB strains had the fastest mycelial growth. Under total darkness conditions, the *R. solani* AG-1-IB strains had the fastest mycelial growth. The above results were consistent with the research results of Sneh et al. [47]. The mycelial growth and sclerotium formation of *R. solani* were significantly affected by temperature and illumination, and suitable temperature and illumination are conducive to *R. solani* mycelial growth and sclerotium formation. Therefore, it can be effectively prevented and controlled according to the biological characteristics of the pathogen. Indeed, illumination conditions control measures can be taken in tobacco seedlings to prevent mycelium of *R. solani* from colonizing the seedlings and to reduce seedling diseases. In addition, this study also found differences in the pathogenicity of different anastomosis groups strains, which was consistent with the research results of Zou et al. [20]. It is speculated that the expression levels of pathogenic genes in the four anastomosis groups strains are inconsistent, leading to differences in pathogenicity. This hypothesis needs to be verified in the next study.

The metabolic phenotype characteristics of microorganisms under different osmotic pressures and pH can reflect their adaptability to the environment. The metabolic phenotypes of different *R. solani* anastomosis group strains on carbon substrate, nitrogen substrate, pH and Osmotic pressure were different. Carbon substrate is the basic nutrient for biological survival, and nitrogen substrate can provide the nitrogen required for the growth and development of organisms [32]. Studies have shown that microorganisms can utilize nutrients in FF microplates and PM microplates, as reported by Wang et al. [48]. Streptomyces can metabolize 77 carbon substances in Biolog FF plates. Wang et al. reported that *Kyushu Fusarium* can efficiently metabolize 69 carbon substrates in the FF microplates and can moderately metabolize 18 carbon substrates [49]. This article found that the *R. solani* strains of four anastomosis groups can metabolize all carbon substrates in FF microplates, indicating that *R.solani* has a stronger ability to metabolize carbon substrates compared to other pathogen. Reducing the addition of carbon sources during seedling cultivation to inhibit the growth of *R. solani*. From previous work, it has been demonstrated that tobacco brown spot pathogen can efficiently metabolize over 60 nitrogen substrates, including L-glutamic acid and L-lysine, in PM3 microplates [50]. The *R. solani* studied in this article can utilize 190 nitrogen substrates, and the nitrogen substrates that can be efficiently metabolized include L-glutamic acid. In addition, this study found that *R. solani* AG-3 strains has stronger Osmotic pressure and pH environment adaptability than other anastomosis groups strains, which is conducive to the *R. solani* AG-3 to survive many adverse environments. It could also be one of the reasons why *R. solani* AG-3 strains are more common than other anastomosis groups strains. *R. solani* AG-3 has a wide range of adaptation to pH, indicating that acidic conditions are more favorable to its growth than alkaline conditions, and it should be a weakly acidophilic pathogen. Therefore, in production, attention should be paid to soil acidity and alkalinity as well as the previous crop planted when planting tobacco in order to minimize the occurrence of diseases. The number of carbon substrates metabolized was highest for the *R. solani* AG-6 strains from tobacco target spot leaves. The reason for this difference is unclear and might be that *R. solani* AG-6 strains contains more genes for metabolizing carbon substrates than the other anastomosis groups strains. More work could be conducted to verify this hypothesis in the next study. Different concentrations of osmotic pressure substances can be applied to control tobacco target spot disease or reduce the degree of harm caused by the disease, which requires further experiments to determine. In addition, the growth environment of tobacco target spot pathogen is complex in the field, and further research on the pathogenicity factors of tobacco target spot pathogen is needed under various factors such as soil type, microorganisms, and host resistance interaction modes in the future.

## Materials and methods

### Origin of *R. solani* strains

The tested strains in different anastomosis groups were identified and stored at Guizhou Provincial Academician Workstation of Microbiology and Health, Guizhou Academy of Tobacco Science. Three strains (J215, J216, J136) of the *R. solani* AG-6 and three strains (B6-8, B7-1, T1-141) of the *R. solani* AG-5 were the first identified new anastomosis groups on tobacco in Guizhou, China. Three strains (AG-31, AG-32, AG-33) of the *R. solani* AG-3. Three strains (LK1, LK2, LK3) of the *R. solani* AG-1-IB were selected as control, which causing tobacco sore shin. The strains information is shown in Table S1.

### Test materials and instruments

The test medium is potato dextrose agar medium (PDA: 200 g of potato, 17 g of agar powder, 20 g of dextrose, 1000 mL of distilled water, autoclaved). Water agar medium (oligotrophic medium, WA: 20 g agar powder, 1000 mL distilled water, autoclaved). Potato dextrose broth medium (PDB: potato 200 g, glucose 20 g, distilled water 1000 mL, autoclaved). The tested tobacco seedlings are K326 tobacco seedlings with good growth and healthy appearance. The reagents and instruments were Biolog FF microplates, Biolog PM microplates, FF-IF inoculum, OmniLog system and 8-channel electric pipette, all purchased from Biolog, U.S.A. D-glucose (Sigma, U.S.A.), yeast nitrogen substrate (Difco, U.S.A.).

### Effect of temperatures to mycelial growth and sclerotium formation of *R. solani*

The test strains were cultured on PDA medium for 3 d, and the PDA plugs with mycelial were made at the edge of the colonies with a sterilized punch with an inner diameter of 6 mm, inoculated into the middle of PDA medium, and incubated in the dark incubators at 5, 10, 15, 20, 25, 30, and 35℃, respectively. Four replicates for each treatment and after 48 h of incubation, the colony diameters were measured. The measured plates were continued to be incubated and observed, and the status of the colony plates was observed daily, and the number of sclerotium was recorded at the beginning of sclerotium formation and 10 d after sclerotium formation.

### Effects of light condition to mycelial growth and sclerotium formation of *R. solani*

The test strains were cultured on PDA medium for 3 d, and the PDA plugs with mycelial were made at the edge of the colonies with a sterilized punch with an inner diameter of 6 mm, inoculated into the middle of PDA medium. The test plates were incubated at 25 °C, continuous illumination, 25 °C, complete darkness and 25 °C, 12 h alternating light and dark, respectively. Four replicates were conducted for each treatment, and after 48 h of incubation, the colony diameters were measured. The method for observing the number of sclerotium formation was the same as above.

### Effect of medium to mycelial growth and sclerotium formation of *R. solani*

The test strains were cultured on PDA medium for 3 d. The PDA plugs with mycelial was made at the edge of the colony with a sterilized punch with an inner diameter of 6 mm and inoculated into the middle of water agar medium (WA), and incubated in the dark incubators at 25 °C. Four replicates were set up for each treatment, and the colony diameters were measured after 48 h of incubation. We observed the status of colony plates every day, and took photos and recorded at the beginning of sclerotium formation, at the growth stage of sclerotium, at the maturity stage of sclerotium, and finally recorded the number of sclerotium.

### Analysis of differences in sclerotium formation of *R. solani* at different anastomosis groups

The test strains were cultured on PDA medium for 3 d. The PDA plugs with mycelial was made at the edge of the colony with a sterilized punch with an inner diameter of 6 mm and inoculate into the middle of the PDA medium, and cultivate them in dark incubators at 25℃. Four replicates were set for each treatment, observe the status of the colony plates every day, take photos at the beginning of the formation of the sclerotia, record the growth period of the sclerotia, take photos at the mature stage of the sclerotia, and finally record the number of sclerotia.

### Pathogenicity of different *R. solani* anastomosis groups

The test strain was incubated on PDA medium for 3 d. The PDA plugs was made at the edge of the colony with a sterilized punch with an inner diameter of 6 mm (standby). The leaves were collected from the 2nd to 3rd leaf position (from the bottom to up) of the 7 ~ 8 leaf stage tobacco plant of K326 variety. The leaves disinfected with 75% alcohol, washed with sterile water in turn, and air-dried. The same wounds were prepared with sterile inoculation needles at symmetrical sites away from the leaf veins. The wounds were inoculated with 6 mm diameter PDA plugs with *R. solani*. and the mycelial surface close to the leaf, and each strain was repeated six times. After inoculation, these leaves were placed in an artificial climate incubator (temperature 28 °C, relative humidity 70%, 12 h of alternating light and dark conditions) and incubated for 24 h. The PDA plugs were removed with a sterile toothpick and the inoculated leaves was observed daily. The time of incidence was recorded, and the incidence of leaves was observed at 3 d, 5 d, 7 d and 9 d after the incidence of each anastomosis group of inoculated leaves, respectively. The diameter of each lesion was measured. The differences in the pathogenicity of each anastomosis group strain were evaluated by the size of the lesion after the inoculation of each anastomosis group strains.

### Phenotype differences in carbon substrate metabolism among different anastomosis groups of *R. solani*

The test strains AG-32, B6-8, J136 and LK3 (one from each anastomosis group) were randomly selected from the above study. The test strains (AG-32, B6-8, J136, LK3) were incubated on PDA medium for 3 d. The PDA plugs with mycelia was made at the edge of the colony with a sterilized punch with an inner diameter of 6 mm. The mycelial suspension was made by reference to the method of Zang et al. [51]. The PDA plugs with mycelial was inoculated into sterilized triangular flasks containing PDB liquid medium, and the triangular flasks were placed in a shaker at 25 °C, 180 rpm shaking for more than 96 h. A sufficient amount of mycelium grows in the triangular flask, the solid medium at the time of inoculation was removed with sterilized toothpicks and forceps, and the remaining pure mycelium was filtered and washed with distilled water until the distilled water was clear. The appropriate amount of cleaned mycelium was transferred to a sterile 2 mL centrifuge tube. Adding the appropriate amount of FF inoculum in 2 mL centrifuge tube. The mycelium was ground into uniform mycelial fragments using a grinder at 2000 rpm. All mycelial fragments were transferred to a sterilized triangular flask and FF inoculum was added to make a mycelial suspension. The concentration of the mycelial suspension was adjusted to 62% T (T is the standard concentration unit of Biolog) [52]. The mixed mycelial suspension was added to the Biolog FF microplates using an 8-channel electric pipette at 100 µL per well. The FF microplates were incubated in an OmniLog incubator at 25 °C for 7 d. Established OmniLog working software and collected data. Using DataAnalysis software to analyze data, convert data to. csv format, and further convert data to. xlsx format. The carbon substrate metabolic phenotypic characteristics of the mycelium were analyzed according to its metabolic profile. Useing HemI (version 1.0.3.3) software to plot the heat map.

### Phenotype differences in nitrogen substrate metabolism among different anastomosis groups of *R. solani*

Prepare mycelium suspension according to the above method and adjust the concentration of the mycelium suspension to 62% T. Adding the mixed mycelial suspension to PM 5, 6 microplates using an 8-channel electric pipette at 100 µ L per well. Incubate PM 5, 6 microplates in OmniLog incubator at 25 °C for 7 d. Established OmniLog working software and collected data. Using DataAnalysis software to analyze data, convert data to. csv format, and further convert data to. xlsx format. The nitrogen substrate metabolism phenotype was characterized according to the metabolic profile of the mycelium. Useing HemI (version 1.0.3.3) software to plot the heat map.

### Phenotype differences in osmotic pressure and Ph metabolism among different anastomosis groups of *R. solani*

Prepare mycelium suspension according to the above method and adjust the concentration of the mycelium suspension to 62% T. The mixed mycelial suspension and PM microplates additive [26] were added to PM 9 and PM 10 microplates using an 8-channel electric pipette at 100 µL per well. The PM 9 and 10 microplates were incubated in an OmniLog incubator at 25 °C for 7 d. The OmniLog working software was set up and data were collected. Using DataAnalysis software to analyze data, convert data to. csv format, and further convert data to. xlsx format. The pH and osmotic pressure metabolic phenotypic characteristics of the mycelium were analyzed according to its metabolic profile. Useing HemI (version 1.0.3.3) software to plot the heat map.

### Electronic supplementary material

Below is the link to the electronic supplementary material.

Supplementary Material 1

## Data Availability

Data are contained within the article and supplementary materials.

## References

[1] Wang HC, Wang J, Chen QL, Wang MS, Hsiang T, Shang SH, Yu ZH (2016). Metabolic effects of azoxystrobin and kresoxi kresoxim-methymethyl against *Fusarium kyushuense* examined using the Biolog FF micro plate. Pestic Biochem Phys.

[2] Sun ML, Shi CH, Xiao BQ (2023). Composition and diversity of phyllospheric microbial community in tobaccoleaves infected by tobacco target spot disease. Tob Sci Technol.

[3] Sun ML, Shi CH, Huang Y, Wang HC, Li JJ, Cai LT (2023). Effect of disease severity on the structure and diversity of the phyllosphere microbial community in tobacco. Front Microbiol.

[4] Liu XR, Zhang J, Wang XJ (2020). Study on the environmental factors and virulence of basidiospore of tobacco target spot in tobacco field. J Yunnan Agricultural Univ (Natural Science).

[5] Fu Y, Wu YH, Mu LX, Zhao XX (2011). Screening of fungicides for tobacco target spot disease in laboratory. Jiangsu Agricultural Sci.

[6] Shew HD, Melton TA (1995). Target spot of tobacco. Plant Dis.

[7] Shew HD, Main CE (1985). *Rhizoctonia* leaf spot of flue-cured tobacco in North Carolina. Plant Dis.

[8] Wu YH, Fu Y, Zhao XX (2013). The anastomosis groups and ITS sequence analysis of *Rhizoctonia Solani* isolates of tobacco target spot. Acta Phytopathol Sin.

[9] Elliott PE, Lewis RS, Shew HD (2008). Evaluation of tobacco germplasm for seedling resistance to stem rot and target spot caused by *Thanatephorus Cucumeris*. Plant Dis.

[10] Hou HH, Sun JP, Liu ZY, Wang XJ, He YS, Wu YH (2018). Identification and anastomosis groups of tobacco target spot disease (*Rhizoctonia Solani* Kühn) in Yunnan tobacco planting areas. J Shenyang Agricultural Uinversity.

[11] Yang CY, Geng CA, Huang XY (2014). Noreudesmane sesquiterpenoids from the leaves of *Nicotiana tabacum*. Fitoterapia.

[12] Sun ML, Wang HC, Guo MY, Cai LT, Liu TT, Lu N (2022). Inhibition activity of four fungicides against tobacco target spot pathogen. Guizhou Agricultural Sci.

[13] Gonzalez M, Pujol M, Metraux JP (2011). Tobacco leaf spot and root rot caused by *Rhizoctonia Solani* Kühn. Mol Plant Pathol.

[14] Wu YH, Zhao YQ, Zhao XX, An MN, Chen J (2012). Identification and biological characteristics of pathogen causing target spot on tobacco. J Shenyang Agricultural Univ.

[15] Zu QX, Zhang YF, Feng YX (2022). Research progress on pathogen biology and comprehensive prevention and control measures of tobacco target spot. Mod Agricultural Sci Technol.

[16] Parmeter JR, Sherwood RT, Platt WD (1969). Anastomosis grouping among isolates of *Thanatephorus Cucumeris*. Phytopathology.

[17] Carling DE, Baird RE, Gitaitis RD (2002). Characterization of AG-13, a newly reported anastomosis group of *Rhizoctonia solani*. Phytopathology.

[18] Ogoshi A (1987). Ecology and pathogenicity of anastomosis and intraspecific groups of *Rhizoctonia Solani* Kühn. Annu Rev Phytopathol.

[19] Xiao YS, Zhong Q, Wu WX, Li SJ, Zhu JZ, Zhong J (2020). Pathogen identification and molecular detection of tobacco target spot in Hunan Province. J Hunan Agricultural Univ (Natural Sciences).

[20] Zou HL. Pathogen identification and biocontrol strain screening of tobacco target spot disease in Hunan. Hunan Agriculture University; 2021.

[21] Qiu MJ, Li YY, Xu TT, Liu HF, Wu CF, Zheng L (2022). Identification and anastomosis group study of the tobacco target spot pathogen in Hubei Province. Chin Tob Sci.

[22] Chen YY, Tan HW, Lu YH (2016). Anastomosis groups of *Rhizoctonia* solani causing tobacco sore shin and target spot in Guangxi. Guangdong Agricultural Sci.

[23] Sun ML, Shi CH, Ju L, Wang HC, Cai LT, Liu TT (2022). First report of target spot caused by *Rhizoctonia Solani* AG-6 in tobacco in China. Plant Dis.

[24] Wang HC, Huang YF, Cai LT, Guo MY, Sun ML, Li F (2023). First report of target spot caused by *Rhizoctonia Solani* AG-5 on tobacco in China. Plant Dis.

[25] Bochner BR, Gadzinski P (2001). Phenotype microarrays for high-throughput phenotypic testing and assay of gene function. Genome Res.

[26] Bochner BR (2003). New technologies to assess genotype–phenotype relationships. Nat Rev Genet.

[27] Wragg P, Randall L, Whatmore AM (2014). Comparison of Biolog GEN III Microstation semi-automated bacterial identification system with matrix-assisted laser desorption ionization-time of flight mass spectrometry and 16S ribosomal RNA gene sequencing for the identification of bacteria of veterinary interest. J Microbiol Meth.

[28] Tohsato, Yukako HM (2008). Phenotype profiling of single gene deletion mutants of E. Coli using Biolog technology. Genome Inf.

[29] Zhang WY. (2017). Study on the effects of red tree leaf litter on microbial diversity based on Biolog-ECO technology. *Shenzhen University*.

[30] Wang HC, Huang YF, Chen XJ (2018). Difference analysis betw een azoxystrobin-sensitive and -resistant isolates of *Alternaria alternata* causing tobacco brown spot in metabolic phenotypic characterization. Acta Phytopathologica Sinica.

[31] Ge Z, Du H, Gao Y (2018). Analysis on metabolic functions of stored rice microbial communities by BIOLOG ECO microplates. Front Microbiol.

[32] Liu TT, Zeng YT, Wang HC, Cai LT, Zhang CQ (2021). Metabolic and microbial community structure analysis in the phyllosphere of tobacco leaves with different maturity during brown spot occurring season. Chin Tob Sci.

[33] Liu C, Xiang LG, Wang HC, Zeng YT, He YF, Yu ZH (2021). Effects of temperature on pathogenicity and metabolic phenotype of tobacco black shank pathogen *Phytophthora nicotianae*. J Plant Prot.

[34] Carling DE. (1996). Grouping in *Rhizoctonia* solani by hyphal anastomosis reaction[M]//*Rhizoctonia* species: taxonomy, molecular biology, ecology, pathology and disease control. Dordrecht: Springer Neth. 37–47.

[35] Woodhall JW, Lees AK, Edwards SG (2007). Characterization of *Rhizoctonia solani* from potato in Great Britain. Plant Pathol.

[36] Mew TW, Rosales AM (1986). Bacterization of rice plants for control of sheath blight caused by *Rhizoctonia solani*. Phytopathology.

[37] Friedl MA, Kubicek CP, Druzhinina IS (2008). Carbon source dependence and photostimulation of conidiation in *Hypocrea Atroviridis*. Appl Environ Microbiol.

[38] Dubey SC, Tripathi A, Upadhyay BK (2014). Diversity of *Rhizoctonia solani* associated with pulse crops in different agro-ecological regions of India. World J Microb Biot.

[39] Muzhinji N, Truter M, Woodhall JW (2015). Anastomosis groups and pathogenicity of *Rhizoctonia solani* and binucleate *Rhizoctonia* from potato in South Africa. Plant Dis.

[40] Xia B, Xu CT, Xu JK (2019). First Report of Target Leaf Spot on Flue-Cured Tobacco by *Rhizoctonia Solani* AG-3 in Sichuan, China. Plant Dis.

[41] Scholte K, Lootsma M (1996). Effects of farmyard manure and green manure crashes on populations of mycophagus soil failure and *Rhizoctonia* stem canker of potato. Pedobiologia.

[42] Virgen CG, Olalde-portugal V, Carling DE (2000). Anastomosisgroups of *Rhizoctonia solani* on potato in central Mexico andpotential for biological and chemical control. AmericanJournal Potato Res.

[43] Misawa T, Kuninaga S (2010). The first report of tomato foot rot caused by *Rhizoctonia Solani* AG-3 PT and AG-2-Nt and its host range and molecular characterization. J Gen Plant Pathol.

[44] Salazar O, Julián MC, Hyakumachi M (2000). Phylogenetic grouping of cultural types of *Rhizoctonia Solani* AG-2-2 based on ribosomal ITS sequences. Mycologia.

[45] Fan TWM, Lane AN, Shenker M, Bartley JP, Crowley D, Higashi RM (2001). Comprehensive chemical profiling of gramineous plant root exudates using high-resolution NMR and MS. Phytochemistry.

[46] Lung SC, Leung A, Kuang R, Wang Y (2008). Phytase activity in tobacco (*Nicotiana tabacum*) root exudates is exhibited by a purple acid phosphatase. Phytochemistry.

[47] Sneh B, Jabaji-Hare S, Neate SM, Dijst G (2013). *Rhizoctonia* species: taxonomy, molecular biology, ecology, pathology and disease control. Springer Sci Bus Media.

[48] Wang YS, Chen YJ, Zhang Y (2012). Isolation, identification and carbon metabolic fingerprinting analysis of four pathogens isolated from postharvest plum fruit. Food Sci.

[49] Wang HC, Wang J, Li LC, Hsiang T, Wang MS, Shang SH (2016). Metabolic activities of five botryticides against *Botrytis cinerea* examined using the Biolog FF microplate. Sci Rep.

[50] Wang HC, Wang J, Chen QY. (2017). Metabolic effects of azoxystrobin and kresoxim-methyl against Fusarium kyushuense examined using the Biolog FF microplate. *Outstanding Papers Collection of the 2017 Academic Annual Meeting of the China Tobacco Society*, *China Tobacco Society*. 2017:3019–3030.

[51] Zang XP, Xu YP, Cai XZ (2010). Establishment of an inoculation method for *Sclerotinia Sclerotiorum* based on mycelium suspension. J Zhejiang University: Agric Life Sci Ed.

[52] Arakawa M, Inagaki K (2014). Molecular markers for genotyping anastomosis groups and understanding the population biology of *Rhizoctonia* species. J Gen Plant Pathol.

