# Supplementary material for: Biological characteristics and metabolic phenotypes of different anastomosis groups of Rhizoctonia solani strains
Source: BMC Microbiol. 2024 Jun 20;24:217. doi: 10.1186/s12866-024-03363-9 (PMC11188240; doi:10.1186/s12866-024-03363-9)
Supplement: Supplementary file 1 — Supplementary Material 1 [file 12866_2024_3363_MOESM1_ESM.docx]

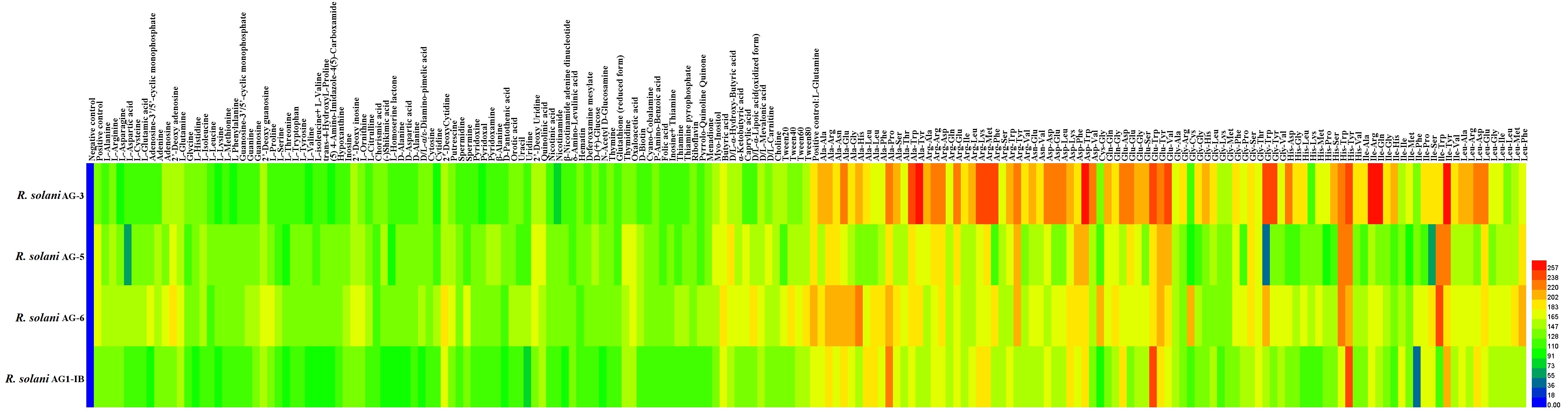


**Figure S1** Heat map of 190 nitrogen sources metabolism abundance of different anastomosis group strains of *Rhizoctonia solani*

Note: The legend of color code from blue to green, and red shades indicate low, moderate, and high utilization of carbon sources, respectively, assessed as arbitrary Omnilog values.

**
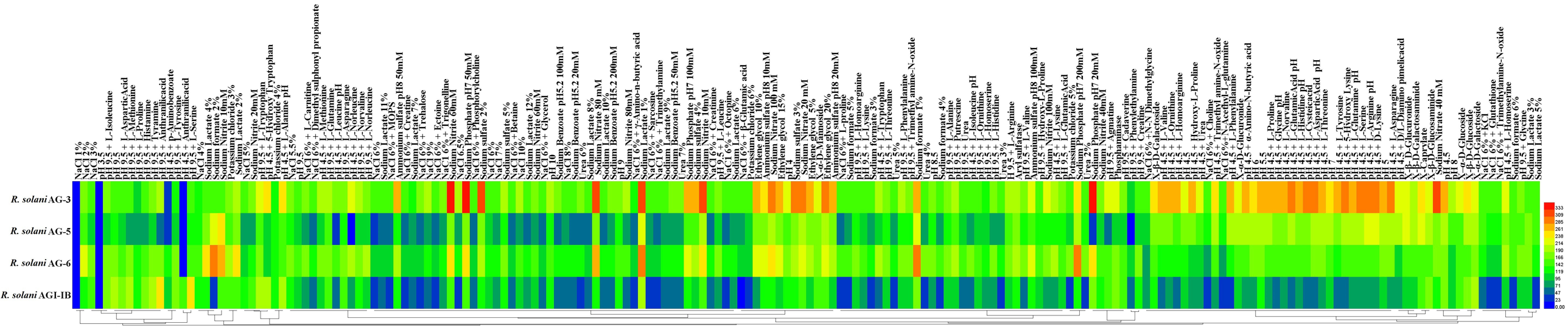
**

**Figure S2** Heat map of 192 pH and osmotic pressure metabolism abundance of different anastomosis group strains of *Rhizoctonia solani*

Note: The legend of color code from blue to green, and red shades indicate low, moderate, and high utilization of carbon sources, respectively, assessed as arbitrary Omnilog values.

**Table S1** Test strains information table

| Anastomosis group | Strain number | Source | Gene accession number |
| --- | --- | --- | --- |
| *R. solani* AG-3 | AG31 | Guizhou Academy of Tobacco Science | OQ711785 |
|  | AG32 |  | OQ711786 |
|  | AG33 |  | OQ711787 |
| *R. solani* AG-5 | B6-8 |  | OL676991 |
|  | B7-1 |  | OL676993 |
|  | T1-141 |  | OL676996 |
| *R. solani* AG-6 | J215 |  | MZ379471 |
|  | J216 |  | MZ379470 |
|  | J136 |  | MZ379468 |
| *R. solani* AG-1-IB | LK1 |  | OQ711782 |
|  | LK2 |  | OQ711783 |
|  | LK3 |  | OQ711784 |
